# Demographics and injuries in assaults from drive-by shootings seen in US emergency departments 1993-2020

**DOI:** 10.5249/jivr.v17i1.1913

**Published:** 2025-01

**Authors:** Randall T. Loder, Faith Kylee Darden

**Affiliations:** ^*a*^ Riley Children’s Hospital and Department of Orthopaedic Surgery, Indiana University School of Medicine, Indian-apolis, Indiana, USA.; ^*b*^ Indiana University School of Medicine, Indianapolis, Indiana, USA.

**Keywords:** Firearm, Drive-by shooting, Demographics, Injury, United States

## Abstract

**Background:**

There has been minimal research on drive-by shootings since the 1990s. It was the purpose of this study to investigate the demographics and injury patterns of drive-by shootings across the entire US using a national database.

**Methods:**

The Inter-University Consortium for Political and Social Research Firearm Injury Surveillance Study 1993-2020 (ICPSR 38574) data for 1993 through 2020 was analyzed using statistical analyses accounting for the stratified and weighted nature of the data.

**Results:**

There were an estimated 63,882 emergency department visits due to drive-by shootings. The drive-by group was younger compared to the no-drive-by group (average age 24.5 years vs. 28.7 years – p less than 10^-4^). Patients injured in drive-by shootings were more prevalent in medium and large size hospitals. There was a lower percentage of White (17.9% vs. 42.3%) and a higher percentage of Hispanic (30.1% vs. 13.1%) peoples in the drive-by group compared to the no-drive-by group (p = 0.0009). The head/neck (14.3% vs. 3.5%) and lower extremity (35.5% vs. 25.5%) were more commonly injured in the drive-by group compared to the no-drive by group (p = 0.0008). While those in the drive-by group were admitted to the hospital more often (43.9% vs. 32.7%), there was no difference in the percentage of fatalities between the two groups (4.4% drive-by, 4.9% no-drive-by).

**Conclusions:**

This study encompasses both rural and urban areas, all races, and both sexes. These national estimates give health care providers and health facility administrators important demographic information. While both drive-by and no-drive-by shootings increased from 2014 onward, the average annual increase was much greater for the drive-by group (22.7%) compared to the non-drive-by group (8.6%). This data provides helpful information that could be useful when analyzing prevention strategies and firearm legislation and their impact on drive-by shootings.

## Introduction

Firearm injuries in the US are a significant public health issue.^1,2^ This is due to the number of deaths^1^ as well as societal costs.^2-4^ Drive-by shootings, which are a subset of firearm injuries, are not well studied. In an older study from Los Angeles 1989 to 1993,^5^ there were 6,327 drive-by shootings with 9,053 people shot at and 590 homicides. There was an increase in these drive-by shootings from 1989 -1991, followed by a decrease from 1991-1993. Innocent bystanders accounted for 47% of the people shot at and 23% of the homicide victims; 94% of the homicide victims were Black or Hispanic. In a recent 2020 study from Detroit,^6^ out of 275 patients≤18 years old with firearm injuries, 47% were due to drive-by shootings. The type of firearm in drive-by shootings is typically a powder firearm^5,6^ but occasionally air propelled firearms are used.^7^

Aside from the Los Angeles studies^5,8-10^ in the 1990s and the Detroit decade 2010 study,^6^ there has been minimal research of drive-by shootings. Furthermore, there is no nationwide study over a long period of time. It was the purpose of this study to renew studies of drive-by shootings and investigate the patterns of injury and demographics in patients sustaining injuries from drive-by shootings across the entire US using a national emergency department (ED) database. Such a US nationwide study has never been published; Thus, this study will be seminal regarding drive-by shootings across the entire US. Such information may be helpful for legislative bodies and firearm policy makers. 

## Materials and Methods

**Data Source**

The Firearm Injury Surveillance Study 1993-2020 (ICPSR 38574) data (https://www.icpsr.umich.edu/web/NACJD/studies/38574), collected by the National Electronic Injury Surveillance System (NEISS), was used for this study. The NEISS data is a statistically weighted and stratified sample of patients with injuries presenting to hospitals having an emergency department (ED) in the United States. The NEISS data is commonly used in injury research and plays a critical role in injury surveillance and national-level injury research.^11^ Further details regarding the NEISS data and the acquisition of the ICPSR/NEISS data and guidelines for its use are available at their respective web sites (ICPSR - www.icpsr.umich.edu, NEISS -www.cpsc.gov/library/neiss.html). The Firearm Injury Surveillance Study data, maintained by the Inter-University Consortium for Political and Social Research in Ann Arbor, MI, USA, is a data set of 47 variables.^12^ These variables include typical demographic variables along with information regarding the injury diagnosis and anatomic locations, hospital disposition, firearm type, and many others. Finally, there is a data column giving narrative comments extracted from the ED medical record for every patient, in essence a mini-history. These narrative comments are entered daily into the NEISS database by the coders at each of the respective NEISS hospitals for the firearm injuries occurring the day before. Using these narrative comments drive-by shootings were found by the authors using the FIND command in Excel (Microsoft Office 365) for the terms driveb, drive-by, drive-b, driveth, drive th, and drive-th. Earlier versions of the Firearm Injury Surveillance Study have been used to study other aspects of firearm injuries by our group^13-15^ but not drive-by shootings. Each year the NEISS adds an additional year into their data sets, with the addition typically lagging behind by 2 or 3 years. In this study, the data for the Firearm Injury Surveillance Study 1993-2020 (ICPSR 38574) was the most recent becoming available on the website on 11 November 2022 when this study began in December 2022. This study of publicly available de-identified data was considered exempt by our local Institutional Review Board.

**Statistical Analysis**

Due to the stratified and weighted nature of the Firearm Injury Surveillance Study data, statistical analyses must account for such a design.^16^ SUDAAN 11.0.01™ software (RTI International, Research Triangle Park, North Carolina, 2013) was therefore used. This calculates an estimated national number (N) of ED visits, along with 95% confidence intervals (CI) of the estimated N. These methods are well described by the NEISS^16^ and the Centers for Disease Control and Prevention.^17^ The reader is referred to these references if further statistical method descriptions are desired. Analyses of percentage changes in the estimated number of ED visits due to drive-by shootings over time were performed using joinpoint regression analysis (Joinpoint Regression Program, Version 4.8.0.1, April 2020; Statistical Research and Applications Branch, National Cancer Institute [https://surveillance.cancer. gov/joinpoint/]). The t-test (2 groups) or ANOVA (3 or more groups) was used to test for differences in continuous variables while the χ2 test was used to study for differences between groups of categorical variables. Aα value of <0.05 imparted statistical significance. It should be noted the chi-square values and corresponding p values calculated by the SUDAAN software takes into account the 95% confidence intervals for the weighted estimates and their effect sizes. That is, when using the weighted estimates a traditional chi-square analysis might result in a highly significant p value (eg. <0.00001) whereas when SUDAAN takes into account the corresponding 95% confidence intervals the p value may > than 0.05.

## Results

From 1993 – 2020 there were 119,783 actual ED visits for injuries due to firearms giving an estimated 3,586,501 [3,164,728 ^–4^,008,274] ED visits. Of these 3.586 million visits, 2,171 actual visits associated with drive-by shootings were found, for an estimated 63,882 [46,625 – 87, 511] visits (1.8% of all firearm injury associated ED visits). From here forward, only the estimated number (N) is given in the manuscript and figures; the actual (n) and estimated number (N) of ED visits are given in the Tables. 

**Comparisons between the drive-by and no-drive-by groups**

Those in the drive-by group were younger compared to the no-drive-by group (average age 24.5 years vs. 28.7 years – p<10^-4^) (Table 1); the drive-by group had a higher percentage of 15 to 24-year-olds (56.5%) compared to the no-drive-by group (37.8%). Patients injured in drive-by shootings were seen less in small hospitals and more in medium and large size hospitals (Figure 1) (p <10^-4^). There was a much lower percentage of Whites (17.9% vs. 42.3%) and a higher percentage of Amerindians (30.1% vs. 13.1%) in the drive-by shooting group with an equal number of Blacks in both groups (49.7% and 43.8% respectively) (Figure 1B) (p = 0.0009). A powder firearm was used in 96.9% of the drive-by and 84.4% of the no-drive-by shooting group (p <10^-4^). The head/neck (14.3% vs 3.5%) and lower extremity (35.5% vs 25.5%) were more commonly injured in the drive-by group compared to the no-drive-by group (Figure 1C) (p = 0.0008). While those in the drive-by group were more commonly admitted to the hospital (43.9% vs 32.7%), there was no difference in the percentage of fatalities in patients reaching the ED between the two groups (4.4% drive-by, 4.9% no-drive-by) (Figure 1D) (p = 0.0005). As would be expected, the incident locale of those injured in drive-by shootings was on the street/highway (Figure 1E) (p = 0.011). Nearly all of those in the drive-by group were shot (99.1%) vs. the no-drive-by group (80.6%) (p <10^-4^). Upon the authors’ review of the individual comments for the 586 patients not shot, revealed that they were injured when falling to the ground to avoid being shot or injured by glass that had shattered during the shootings. The remaining results are shown in Table 1.

**Table 1:** Firearm injuries due to drive-by shootings or not.

Variable	All Firearm Injuries					Drive-By Shooting					No-Drive By-Shooting					p value
	n	N	L95%CI	U95%CI	%	n	N	L95%CI	U95%CI	%	n	N	L95%CI	U95%CI	%	
**All**	**119,783**	**3,586,501**			**100.0**	**2,171**	**63,882**	**46,625**	**87,511**	**1.8**	**117,612**	**3,522,620**	**3,498,990**	**3,520,868**	**98.2**	**-**
**Age (average)**			**28.6**					**24.5**					**28.7**			**<10^-4^**
**Median/interquartiles**			**24.5 [18.3, 35.1]**					**20.1 [17.4, 27.7]**					**24.6 [18.4, 35.2]**			
**Age group (years)**																**<10^-4^**
**0 to 4**	1,061	27,957	22,236	35,506	0.8	44	454	236	869	0.7	1,017	27,503	22,193	34,874	0.8	
**5 to 9**	2,535	85,187	67,785	108,312	2.4	47	653	422	1,016	1.0	2,488	84,534	67,282	107,792	2.4	
**10 to 14**	7,657	242,340	196,540	301,983	6.8	155	3,072	2,370	3,973	4.8	7,502	239,268	193,744	298,366	6.8	
**15 to 24**	48,306	1,368,804	1,311,942	1,445,719	38.2	173	36,087	33,008	39,147	56.5	47,133	1,332,717	1,276,950	1,408,343	37.8	
**25 to 34**	29,856	880,396	830,275	945,043	24.5	467	14,654	12,508	17,069	22.9	29,389	865,742	816,543	929,267	24.6	
**35 to 44**	15,237	477,367	454,768	507,849	13.3	170	5,337	4,338	6,554	8.4	15,067	472,030	449,839	501,973	13.4	
**45 to 54**	7,991	256,433	242,089	275,085	7.1	62	2,080	1,578	2,741	3.3	7,929	254,353	240,243	273,003	7.2	
**55 to 64**	3,680	126,717	116,203	140,232	3.5	33	1,038	709	1,514	1.6	3,647	125,679	112,724	139,143	3.6	
**65+**	2,537	97,063	82,848	115,127	2.7	17	455	217	945	0.7	2,520	96,608	82,077	114,837	2.7	
**Hospital Size**																**<10^-4^**
**Small**	7,531	591,709	424,642	807,680	16.5	39	3,353	1,667	6,567	5.2	7,492	588,356	423,067	801,748	16.7	
**Medium**	10,830	31	398,819	390,929	0.0	326	17,822	8,356	31,858	27.9	10,504	663,962	380,091	1,086,024	18.8	
**Large**	17,852	1,010,771	548,735	1,650,149	28.2	407	22,236	8,471	41,581	34.8	177,445	988,535	539,313	1,609,837	28.1	
**Very large **	77,255	1,266,322	861,478	1,739,812	35.3	1,165	19,142	10,394	30,989	30.0	760,909	1,247,180	849,304	1,711,993	35.4	
**Childrens’**	6,315	35,915	23,671	54,156	1.0	234	1,329	690	2,543	2.1	6,081	34,586	22,897	52,135	1.0	
**Sex**																**0.01**
**Male**	103,434	3,077,017	3,041,355	3,110,917	85.8	1,763	52,985	51,974	53,923	82.9	101,671	3,024,032	2,988,579	3,057,606	85.9	
**Female**	16,312	508,641	474,741	544,303	14.2	408	10,897	9,959	11,908	17.1	15,904	497,744	464,170	533,197	14.1	
**Race**																**0.0009**
**White**	29,724	1,179,470	963,534	1,407,137	41.9	292	9,607	7,888	11,602	17.9	29,432	1,169,863	953,465	1,397,457	42.3	
**Black**	50,999	1,237,020	926,637	1,565,144	43.9	1,027	26,670	17,510	35,868	49.7	49,972	1,210,350	906,496	1,532,009	43.8	
**Hispanic/Native American**	9,575	378,833	204,198	664,982	13.5	389	16,133	8,591	26,412	30.1	9,186	362,700	195,887	636,012	13.1	
**Asian**	864	21,204	10,984	40,558	0.8	51	1,252	354	4,250	2.3	813	19,952	10,775	37,022	0.7	
**Shot**																**<10^-4^**
**Yes**	1,000,289	2,902,698	2,809,306	2,987,197	80.9	21,143	63,296	62,930	63,524	99.1	98,146	2,839,402	2,747,996	2,922,366	80.6	
**No**	19,494	683,803	599,304	777,195	19.1	28	586	358	952	0.9	19,466	683,217	600,254	774,624	19.4	
**Intent of injury**																**<10^-4^**
**Unknown**	9,746	264,949	213,755	327,448	7.4	16	496	204	1,195	0.8	9,730	264,444	213,471	326,195	7.5	
**Unintentional**	26,057	1,017,487	838,524	1,217,976	28.4	12	263	102	664	0.4	26,045	1,017,224	838,384	1,217,065	28.9	
**Assault**	78,001	2,097,278	1,854,580	2,326,922	58.5	2,143	63,123	62,438	63,486	98.8	75,858	2,033,155	1,791,605	2,264,692	57.7	
**Suicide**	4,731	165,344	119,072	228,460	4.6	0	0.00	460	0	0.0	4,731	165,344	118,712	228,970	4.7	
**Law enforcement**	1,248	42,452	31,561	57,025	1.2	0	0.00	77	0	0.0	1,248	42,452	31,351	57,066	1.2	
**Incident locale**																**0.011**
**Unknown**	54,059	1,539,763	1,357,849	1,726,900	42.9	553	16,904	10,815	24,812	26.5	53,506	1,522,769	1,346,698	1,703,539	43.2	
**Home/apt**	26,736	938,897	785,802	1,110,022	26.2	463	12,282	10,981	13,696	19.2	25,913	926,615	773,567	1,098,001	26.3	
**School/recreation**	2,625	104,273	85,717	126,603	2.9	44	1,311	856	2,006	2.1	2,581	102,962	84,543	125,405	2.9	
**Street/highway**	22,071	545,546	412,448	711,920	15.2	804	24,486	19,414	29,993	38.3	21,267	521,060	389,602	687,263	14.8	
**Other property**	14,506	450,140	376,224	536,182	12.6	306	8,882	7,072	11,058	13.9	14,200	441,258	368,466	526,279	12.5	
**Farm**	146	7,972	50,211	12,194	0.2	1	17	0	121	0.0	145	7,955	5,284	12,329	0.2	
**Firearm Type**																**<10^-4^**
**Powder**	105,069	3,035,266	2,893,230	3,152,534	84.6	2,115	61,915	60,860	62,611	96.9	102,954	2,973,351	2,832,186	3,090,042	84.4	
**Non-powder**	14,714	551,235	433,967	693,271	15.4	56	1,967	1,271	3,022	3.1	14,658	549,268	432,578	690,434	15.6	
**Anatomic area injured**																**0.0008**
**Head/neck**	32,636	1,029,764	969,937	1,091,663	29.3	313	9,008	8,016	10,098	14.3	32,323	120,756	960,841	1,082,457	3.5	
**Upper trunk**	19,173	534,391	483,034	589,984	15.2	374	11,656	10,470	12,948	18.5	18,799	522,735	471,956	578,025	15.1	
**Lower trunk**	15,045	407,948	370,455	448,908	11.6	343	9,856	8,849	10,955	15.6	14,702	398,092	361,049	438,442	11.5	
**Upper extremity**	19,180	641,201	583,299	703,618	18.2	342	10,191	8,987	11,523	16.2	18,838	631,010	573,533	692,731	18.3	
**Lower extremity**	31,335	904,785	863,339	947,421	25.7	766	22,359	20,832	23,935	35.5	30,569	882,426	840,606	925,600	25.5	
**Major diagnosis**																**<10^-4^**
**Puncture**	29,964	865,069	662,369	1,105,709	24.6	870	27,126	19,293	35,586	42.6	29,094	837,943	643,819	1,068,421	24.2	
**Laceration**	11,728	405,611	329,248	496,865	11.5	129	3,806	1,465	9,325	6.0	11,599	401,805	328,133	489,261	11.6	
**Foreign body**	10,556	375,923	289,808	483,836	10.7	162	3,996	1,592	9,471	6.3	10,394	371,927	287,678	476,813	10.8	
**Fracture**	10,498	317,863	250,721	400,731	9.0	175	4,760	3,414	6,580	7.5	10,323	313,103	246,878	394,866	9.1	
**Internal organ injury**	7,171	206,429	166,561	255,299	5.9	77	2,280	1,261	4,070	3.6	7,094	204,149	164,931	252,064	5.9	
**Contusion/abrasion**	6,079	203,462	175,716	235,227	5.8	78	2,491	1,892	3,274	3.9	6,001	200,971	173,575	232,701	5.8	
**Strain/sprain**	601	22,759	18,663	27,819	0.6	3	108	38	312	0.2	598	22,651	18,326	28,007	0.7	
**Concussion**	485	15,149	11,973	19,015	0.4	0	0	0	0	0.0	485	15,149	12,102	19,017	0.4	
**Hematoma**	429	12,842	10,212	16,198	0.4	2	61	6	401	0.1	427	12,781	10,373	15,905	0.4	
**Other**	40,459	1,096,259	900,061	1,313,822	31.1	663	19,066	13,127	26,293	29.9	39,796	1,077,193	885,164	1,290,057	31.2	
**Disposition from ED**																**0.0005**
**Release**	66,362	2,206,630	1,993,611	2,407,025	62.2	1,057	32,963	29,719	36,182	51.7	65,305	2,173,667	1,963,381	2,371,461	62.4	
**Admit**	46,034	1,169,043	986,515	1,367,281	32.9	1,028	27,997	24,856	31,217	43.9	45,006	1,141,046	961,478	1,336,451	32.7	
**Death**	6,145	172,941	150,461	198,368	4.9	80	2,774	2,027	3,773	4.4	6,065	170,167	147,759	195,850	4.9	
**Perpetrator of the injury**																**<10^-4^**
**Unknown**	60,596	1,564,219	1,377,575	1,756,310	43.6	964	27,276	20,698	34,285	42.7	59,632	1,536,943	1,354,447	1,724,675	43.6	
**Stranger**	17,232	495,281	427,870	571,688	13.8	424	12,599	9,985	15,696	19.7	16,808	482,682	416,374	557,631	13.7	
**Self**	21,519	859,276	706,182	1,033,630	24.0	2	33	6	141	0.1	21,517	859,243	705,229	1,034,593	24.4	
**Friend/acquaintance**	6,915	230,100	200,844	263,249	6.4	29	1,279	754	2,153	2.0	6,886	228,821	199,733	262,083	6.5	
**Spouse/ex**	720	25,284	20,802	30,485	0.7	0	0	0	0	0.0	720	25,284	20,783	30,647	0.7	
**Other relative**	2,651	95,878	78,903	116,561	2.7	1	62	95,816	2,650	3.3	2	92,003	79,611	117,303	2.6	
**Other/not seen**	10,150	316,463	265,042	376,583	8.8	751	22,633	293,830	9,399	9.9	7	298,003	262,435	372,693	8.5	

n = actual number of ED visits, N = national estimate of ED visits, L95%CI = lower 95% confidence interval of the N estimate, U95%CI = upper confidence interval of the N estimate

**Figure 1:**
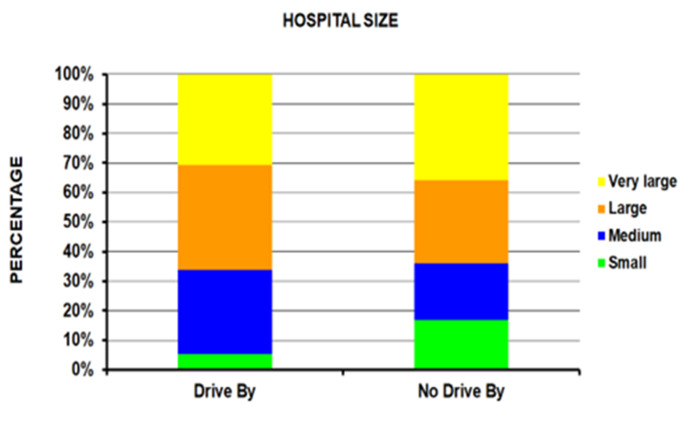
Differences between drive-by and no-drive-by groups. Figure 1A: By hospital size (p < 10^-4^).

**Figure 1B:**
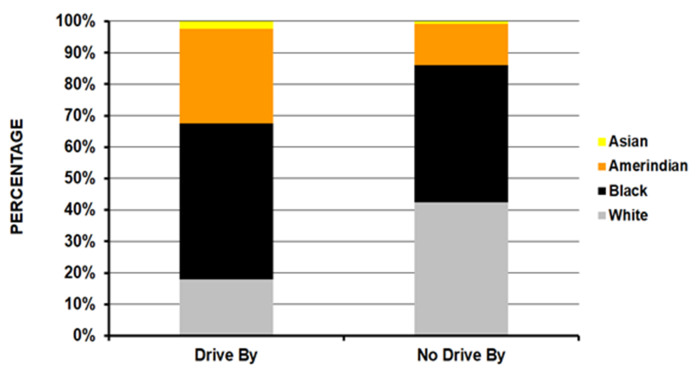
By race of the injured patient (p = 0.0009).

**Figure 1C:**
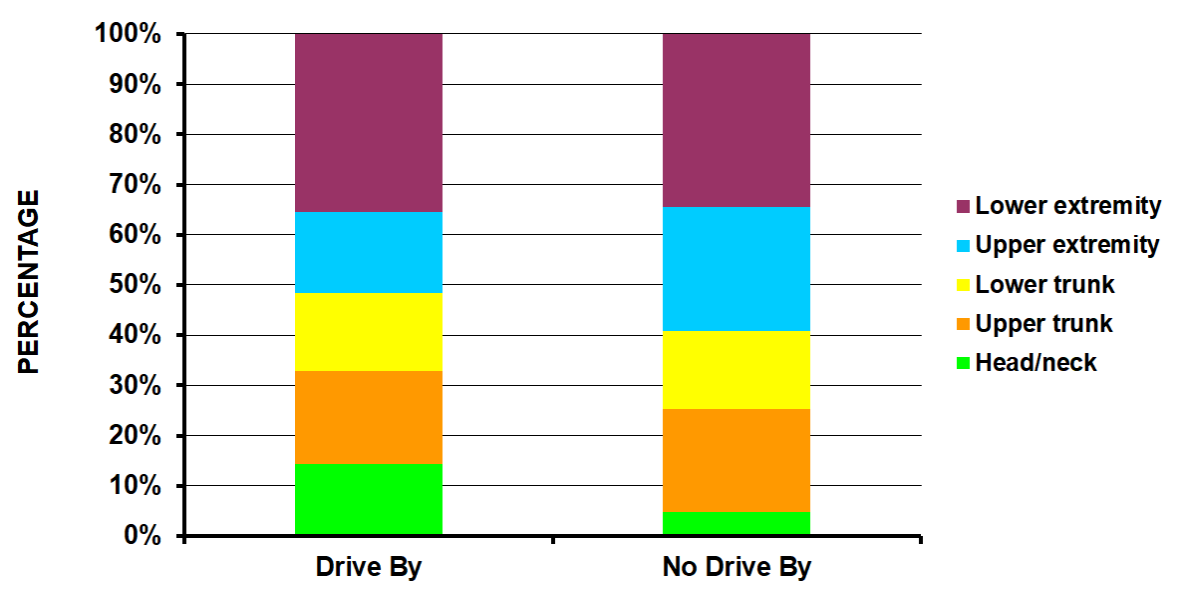
By anatomic area of injury (p = 0.0008).

**Figure 1D:**
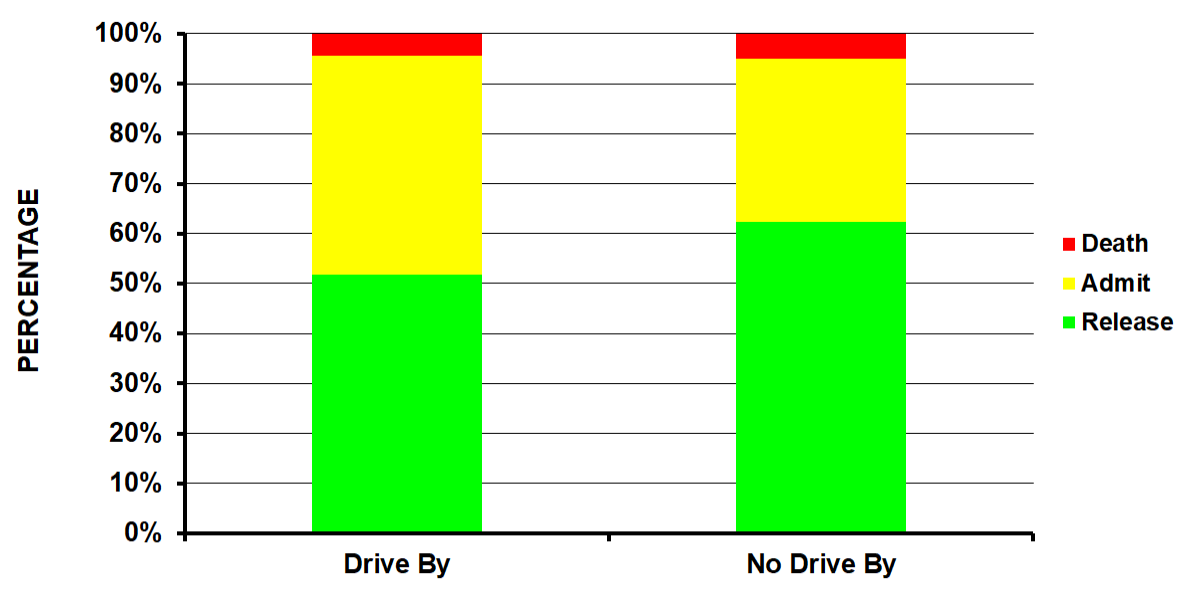
By disposition from the ED (p = 0.0008).

**Figure 1E:**
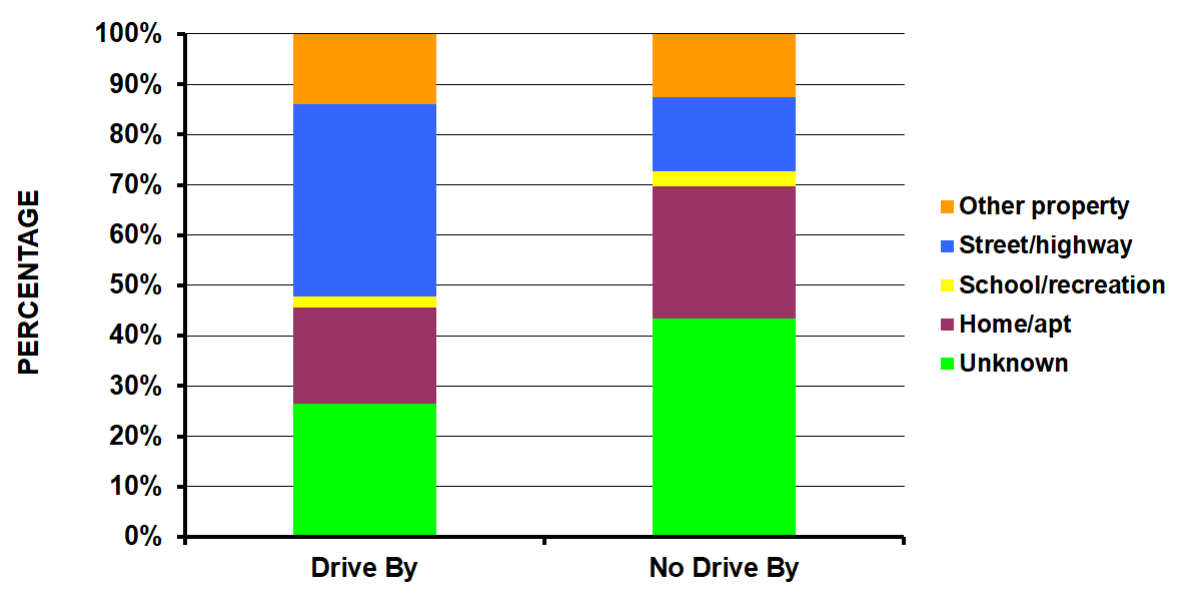
By incident locale (p = 0.011).

There was a decrease in the number of ED visits for firearm injuries from 1993-1999, with a plateau from 1999 -2013. From 2014 through 2020 there was an increase in the number of ED visits for firearm injuries, but at a much higher rate in the drive-by group compared to the no-drive by group (Figure 1F) (p = 0.0008). Joinpoint regression analyses demonstrated an average annual percentage increase from 2014 through 2020 of 22.7% [13.1 - 33.1%] in the drive by and 8.6% [5.7 - 11.5%] in the no-drive by group. 

**Figure 1F:**
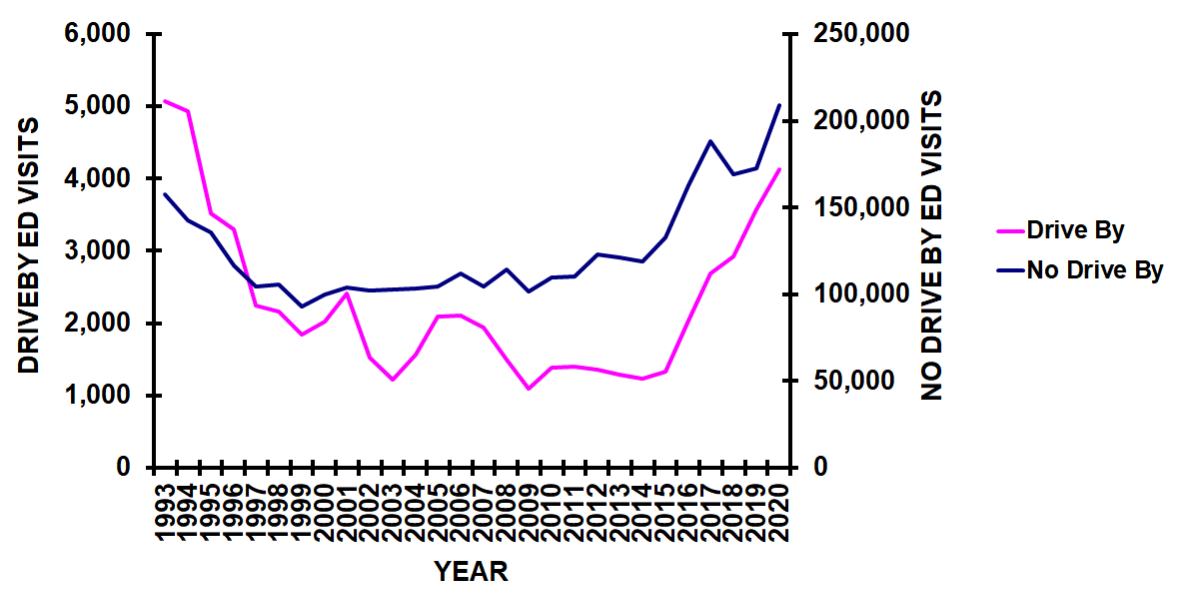
By year of injury (p = 0.0008).

**Analyses within the drive-by group**

Analyses were performed by sex, race, age, disposition from the ED, type of firearm, and anatomic location of injury. There were no differences by sex in any of these parameters. By race, there was a difference in the average age of the injured patients (26.6 years White, 25.6 years Black, and 21.7 years Hispanic/Native American – p <10^-4^). Powder firearms were involved in drive-by shootings in 91.7% of the White, 98.4% of the Black, and 99.0% of the Hispanic/Native American patients (p = 0.018). When analyzing by age (<13 years old vs. ≥ 13 years old) (Table 2) there were differences by sex and incident locale. Females comprised 42.4% of those < 13 years old and 16.3% of those ≥ 13 years old (p = 0.0032). The majority of those injured at home were < 13 years old (56.2%) compared to those ≥ 13 year olds (18.1%).

**Table 2:** Differences in demographic variables in firearm injury drive by shootings between those <13 years and those ≥ 13 years of age

	< 13 years old					13 years or older					p value
	n	N	L95%CI	U95%CI	%	n	N	L95%CI	U95%CI	%	
**All**	131	1,811	0	0	2.8	2,037	62,019	0	0	97.2	-
**Sex**											**0.0032**
**Male**	70	1,043	805	1,263	57.6	1,691	51,907	50,893	52,846	83.7	
**Female**	61	768	548	1,006	42.4	346	10,112	9,173	11,126	16.3	
**Race**											**0.16**
**White**	10	235	90	526	15.4	282	9,372	7,081	11,365	18.4	
**Black**	55	633	423	866	41.5	970	26,003	15,402	35,184	51.1	
**Hispanic/Native American**	30	656	393	948	43.0	359	15,477	7,640	25,248	30.4	
**Disposition**											**0.59**
**Release**	59	924	2,575	4,069	51.0	997	32,022	26,679	32,470	51.8	
**Admit**	63	754	2,104	3,364	41.6	963	27,208	22,294	28,057	44.0	
**Death**	9	133	205	1,059	7.3	71	2,641	1,766	3,350	4.3	
**Firearm Type**											**0.32**
**Powder**	125	1,686	5,138	6,386	93.1	1,987	60,177	54,688	56,218	97.0	
**Non-powder**	6	125	131	1,379	6.9	50	1,842	1,095	2,625	3.0	
**Incident locale**											**0.0001**
**Unknown**	19	209	429	1,267	11.5	534	16,695	9,772	22,788	26.9	
**Home/apartment**	71	1,017	3,035	4,256	56.2	389	11,213	9,187	11,652	18.1	
**School/recreation**	7	97	122	936	5.4	37	1,214	716	1,754	2.0	
**Street/highway**	27	34	1,450	1,127	1.9	777	24,037	17,538	27,281	38.8	
**Other property**	7	39	43	446	2.2	300	8,860	6,511	10,207	14.3	
**Anatomic area injured**											**0.099**
**Head/neck**	33	592	1,379	3,070	33.0	280	8,416	6,909	8,724	13.7	
**Upper trunk**	25	305	746	1,583	17.0	348	11,334	9,368	11,658	18.5	
**Lower trunk**	15	135	239	956	7.5	326	9,686	8,005	9,973	15.8	
**Upper extremity**	16	136	235	986	7.6	326	10,055	8,181	10,510	16.4	
**Lower extremity**	39	625	1,625	2,989	34.9	727	21,734	18,685	21,501	35.5	

n = actual number of ED visits, N = national estimate of ED visits, L95%CI = lower 95% confidence interval of the N estimate, U95%CI = upper confidence interval of the N estimate

## Discussion

There are several similarities as well as differences in our findings compared to previous publications. The average age of the drive-by shooting patients in this study was 24.5 years; there are no studies that give an average overall age of the patients, as they only specifically analyze certain age groups^6,8,9^ or particular injuries.^7^ The racial composition of the drive-by cohort was 17.9% White, 49.7% Black, 30.1% Amerindian (Hispanic/Native American), and 2.3% Asian. This is much different than the no-drive-by firearm injury cohort (Figure 1B) as well as the general US population. The US racial mix in 1990 was 75.6% White, 11.7% Black, 9.0% Hispanic, and 2.7% Asian.^18^ In 2020, it was 74.7% White, 12.0% Black, 16.8% Hispanic, and 4.9% Asian.^19^ This greater proportion of drive-by shooting victims being Black or Hispanic was noted in earlier Los Angeles studies.^5-10^ This present study covers the entire US. 

Racial disparity in firearm violence victims is well known. Kravitz-Wirtz^20^ in a prospective study of children studied at ages 1, 3, 5, 9, and 15 years and spanning the years 1999–2017 found that overall 2–18% of youth resided within 600 m of a gun homicide that occurred in the past year. Black and Hispanic youth were 3–7 times more likely, depending on the exposure radius, to experience a past-year gun homicide than white youth and also experienced incidents more recently and closer to home. In another study, Martin et al.^21^ studied children 5 to 17 years of age during both the pre-COVID and pandemic periods. They found that exposure to firearm violence exposure was lowest among White children and highest among Black children, who experienced 4.44 times greater neighborhood firearm violence exposure. The pandemic increased exposure 27% White children with pandemic effects even greater for nearly all non-White categories.

There are multiple key findings from this study. The type of firearm used in the drive-by-shootings was nearly always a powder type (96.9%), while non-powder firearms (BB guns, other air/gas powered firearms) were used in 15.6% of the non-drive-by shooting group. This would indicate that restriction of non-powder firearms will have little impact on the occurrence of drive-by shootings. Another key finding is that patients involved in drive-by shootings had more serious injuries than non-drive by shootings, as the hospital admission rate increased 11% (43.9% compared to 32.9%) (Table 1). This 10% increase demonstrates that firearms intentionally directed and discharged towards an individual are likely to cause more harm than an accidental discharge, as 57.7% of the non-drive-by shootings were intentional and 28.9% unintentional, compared to 98.8% of the drive-by shootings being intentional and 0.1% being unintentional. While this may seem obvious, it clearly indicates a potential avenue for injury prevention – reducing the intentionality of such shootings by whatever means possible (education, psychological mechanisms of stress reduction, minimizing firearm access to drug dealers and gangs to name a few).

There are also several important findings in this study within the drive-by shooting group. First, those<13 years old injured in drive-by shootings were injured in their home 56% of the time, compared to 18.1% in those≥ 13 years old. This likely means that those<13 years old were innocent bystanders with no involvement in any gang or illegal/drug related activities.^5,10^ Small children being caught in the crossfire of gang violence was described in 1994^8^ and our study supports this finding. The second important finding is the rapid increase in drive-by shootings compared to other firearm associated injuries beginning in 2014. While both drive-by and no-drive-by shootings increased from 2014, the average annual increase was much greater in the drive-by group (22.7%) compared to the no-drive by group (8.6%). These findings are likely due to multifactorial issues. It has been stated that the increase in firearm injury related violence in the US was due to the COVID 19 pandemic,^22,23^ yet the increase began in 2014, far before the 2020 COVID pandemic. However the pandemic did increase exposure to firearm violence for all children, especially those of color^21^ as discussed above. Thus, further investigation into the principal issues leading to this increase in drive-by shootings is warranted.

**Prevention**

It must be noted that there are no studies of particular prevention strategies focused directly on drive-by shootings. Thus the discussion below is of general prevention strategies focused on firearm violence. The demographic findings from this study may assist in the design of prevention initiatives/programs by demonstrating the populations most at risk for injuries due to drive-by shootings. Active prevention strategies should be focused on a particular population with a high risk of sustaining these injuries. Gun violence typically clusters in certain areas^6,24,25^ and perhaps directed education/warnings to people living in these high-risk areas might increase the awareness of risk for drive-by shootings leading to them becoming more cautious in their whereabouts.^6^ However, this will not be enough, as there are cases in the media of patients being shot/killed by drive by shooters while in their homes.^26,27^

Possible passive prevention strategies deployed within the community such as gun buy-back programs and others may be effective options, but they need to be deployed in culturally sensitive and trauma informed manners, address social determinants of health, be appropriately funded, and have proper personnel training.^6,28,29^ However, the efficacy of gun buyback programs is mixed,^30,31^ with one study stating there is clearly no effect of gun buyback programs on firearm related crimes.^32^ Finally, community-based interventions, such as the Cure Violence programs have shown some promise,^33^ Global, 2022 #2519. These programs train carefully selected community partners and local credible messengers to detect and interrupt conflict, promote safer and healthier behaviors and life directions among high-risk individuals, and build healthy social norms.^34^ There have been many successes^29,33,34^ while other studies demonstrate mixed results.^31,35,36^ One promising approach combines both the Curve Violence along with increased police patrol in violence hot spots^37^ supported by another study in Baltimore^33^ where a focus on “bad guys with guns” from law enforcement resulted in 13% reduction in homicides and 19% reduction in non-fatal shootings. However none of these strategies have focused specifically on drive-by shootings.

In a 1987 study of Los Angeles county homicides,^38^ drive-by shootings were highly associated with gangs and accounted for 35% of all homicides in Los Angeles county. A gang truce demonstrated a decrease in the number of gunshot wound patients.^39^

However, this data is nearly 40 years old; can similar results be seen today? This is an unanswered question, but clearly cease fires are a step in the right direction.^40^ More recent data should be collected to investigate current trends and prevalence of gang-related firearm injuries in drive-by shootings.

Limitations of the study must be acknowledged. As this study only analyzes patients seen in EDs, those visiting other health care providers (eg. urgent care centers) are not captured in this data; however, it is likely that any serious firearm injury would be seen in an ED. Next, regional specific analyses could not be done due to the de-identified nature of each hospital in the NEISS sample. Differences by region would be interesting to study,^41^ especially those having stricter gun control laws compared to others, but unfortunately that is not possible due to the de-identified status of each NEISS hospital. It is also possible that some of the patients in this data set were injured in a drive-by shooting but did not convey that information to the ED personnel and would thus not be captured; such number is unknown. Next, as this is an ED focused database, information on the length of stay for those admitted to the hospital, treatment course, and treatments needed (ie. operative, non-operative, etc.) are not available. Finally, while the accuracy of the NEISS data could be questioned, previous firearm studies using NEISS data^42,43^ have demonstrated > 90% accuracy. Finally, the NEISS is a well-recognized database used for ED injury research and has a very high agreement with the ICD injury and diagnosis codes with a κ of 0.87, indicating almost perfect agreement.^11^ The best practice for using NEISS data should include manual review of case narratives^11^ which was done in this study. 

## Conclusion

This study encompasses both rural and urban areas, all races, and both sexes. Such data can give health care providers and health facility administrators important demographic information regarding drive-by shootings. This data serves as baseline information and hopefully will be helpful when analyzing future changes in drive-by shootings regarding effectiveness of prevention strategies and trends following firearm legislation (either for or against further firearm restriction).

**Authors' contributions**

RTL conceived and designed the study, RTL and FKD collected the data, RTL performed statistical analyses, RTL and FKD prepared the original manuscript, RTL and FKD participated in manuscript reviews and approved the final manuscript.
